# Timing-dependent effects of transcranial direct current stimulation with mirror therapy on daily function and motor control in chronic stroke: a randomized controlled pilot study

**DOI:** 10.1186/s12984-020-00722-1

**Published:** 2020-07-20

**Authors:** Wan-wen Liao, Wei-chi Chiang, Keh-chung Lin, Ching-yi Wu, Chien-ting Liu, Yu-wei Hsieh, Yun-chung Lin, Chia-ling Chen

**Affiliations:** 1 Department of Occupational Therapy and Graduate Institute of Behavioral Sciences, College of Medicine, Chang Gung University, 259 Wen-hwa 1st Road, Taoyuan City, Taiwan; 2 Department of Occupational Therapy, I-Shou University, Kaohsiung, Taiwan; 3 School of Occupational Therapy, College of Medicine, National Taiwan University, Taipei, Taiwan; 4 Division of Occupational Therapy, Department of Physical Medicine and Rehabilitation, National Taiwan University Hospital, Taipei, Taiwan; 5 Healthy Aging Research Center, Chang Gung University, Taoyuan, Taiwan; 6 Department of Physical Medicine and Rehabilitation, Chang Gung Memorial Hospital, Linkou, Taiwan; 7 Department of Rehabilitation, Taipei Tzu Chi Hospital, The Buddhist Tzu-Chi Medical Foundation, Taipei, Taiwan; 8 Kaohsiung Chang Gung Memorial Hospital, Kaohsiung, Taiwan; 9 Graduate Institute of Early Intervention, College of Medicine, Chang Gung University, Taoyuan, Taiwan

**Keywords:** Timing-dependent effect, Transcranial direct current stimulation, Mirror therapy, Stroke, Activities of daily living, Upper extremity kinematics

## Abstract

**Background:**

The timing of transcranial direct current stimulation (tDCS) with neurorehabilitation interventions may affect its modulatory effects. Motor function has been reported to be modulated by the timing of tDCS; however, whether the timing of tDCS would also affect restoration of daily function and upper extremity motor control with neurorehabilitation in stroke patients remains largely unexplored. Mirror therapy (MT) is a potentially effective neurorehabilitation approach for improving paretic arm function in stroke patients. This study aimed to determine whether the timing of tDCS with MT would influence treatment effects on daily function, motor function and motor control in individuals with chronic stroke.

**Methods:**

This study was a double-blinded randomized controlled trial. Twenty-eight individuals with chronic stroke received one of the following three interventions: (1) sequentially combined tDCS with MT (SEQ), (2) concurrently combined tDCS with MT (CON), and (3) sham tDCS with MT (SHAM). Participants received interventions for 90 min/day, 5 days/week for 4 weeks. Daily function was assessed using the Nottingham Extended Activities of Daily Living Scale. Upper extremity motor function was assessed using the Fugl-Meyer Assessment Scale. Upper extremity motor control was evaluated using movement kinematic assessments.

**Results:**

There were significant differences in daily function between the three groups. The SEQ group had greater improvement in daily function than the CON and SHAM groups. Kinematic analyses showed that movement time of the paretic hand significantly reduced in the SEQ group after interventions. All three groups had significant improvement in motor function from pre-intervention to post-intervention.

**Conclusion:**

The timing of tDCS with MT may influence restoration of daily function and movement efficiency of the paretic hand in chronic stroke patients. Sequentially applying tDCS prior to MT seems to be advantageous for enhancing daily function and hand movement control, and may be considered as a potentially useful strategy in future clinical application.

**Trial registration:**

ClinicalTrials.gov Identifier: NCT02827864. Registered on 29th June, 2016.

## Introduction

Stroke remains one of the leading causes of long-term disability [1]. Most stroke patients have difficulties performing every day activities due to paresis of upper limbs, which results in impaired activities of daily living (ADL) and reduced quality of life [2, 3]. Identifying strategies that can facilitate functional recovery is thus an important goal for stroke rehabilitation. In recent years, several neurorehabilitation approaches have been developed to augment functional recovery, for example repetitive, task-oriented training and non-invasive brain stimulation (NIBS) [4, 5]. Repetitive, task-oriented training emphasizes repetitive practice of task-related arm movements to facilitate motor relearning and restore correct movement patterns [6]. On the other hand, non-invasive brain simulation aims to maximize brain plasticity by externally applying electrical stimulation to modulate cortical excitability [7]. Since these two types of approaches individually have been shown to improve stroke recovery, it has been proposed that a synergistic approach that combines both of them may further augment overall treatment effects [8, 9].

Mirror therapy (MT) is one type of repetitive task-oriented training that has been widely used in clinical and research settings [10]. During MT training, a mirror is positioned in between the paretic and non-paretic arm. The paretic arm is behind the mirror and participants can only see the non-paretic arm and its mirror reflection. Participants are required to focus their attention on the mirror reflection and imagine it is the paretic arm while performing bilateral movements as simultaneously as possible. This mirrored visual feedback is hypothesized to restore the efferent-afferent loop that is damaged after stroke and facilitate re-learning of correct movement patterns [11]. MT has been demonstrated to reduce arm impairment and improve sensorimotor function and quality of life in individuals with stroke [10–13].

Transcranial direct current stimulation (tDCS) is a commonly used NIBS technique in stroke rehabilitation. tDCS applies weak direct current to the scalp to modulate brain excitability [14]. This weak direct current gradually changes neural membrane potentials to facilitate depolarization (excitation) or hyper-polarization (inhibition) of the neurons to enhance plasticity of the brain [15]. tDCS has been demonstrated to modulate neural networks and enhance motor learning in stroke patients [7, 16–18]. Although tDCS can be used alone, it is often combined with other rehabilitation approaches to boost responses of the brain to therapies [8, 19, 20]. A recent meta-analysis further showed that combining tDCS with rehabilitation interventions could produce greater treatment effects on recovery of motor function than tDCS alone in stroke patients [21].

Combining tDCS with MT is a potentially promising approach to not only augment neural responses of the brain but also increase treatment benefits of MT. Nevertheless, one crucial factor that needs to be considered when combining tDCS with MT is the timing of tDCS [22]. tDCS can be applied prior to MT (i.e., offline tDCS) or concurrently with MT (i.e., online tDCS). To our knowledge, only two studies have examined the synergistic effects of combined tDCS with MT in chronic stroke patients [23, 24]. Cho et al. (2015) applied tDCS prior to MT or motor training without mirror reflection. They found significant improvements in manual dexterity and grip strength in the combined tDCS with MT group, suggesting that sequentially applying tDCS prior to MT could improve motor function. By contrast, Jin et al. (2019) delivered tDCS prior to or concurrently with MT and found advantageous effects on hand function in the concurrent tDCS with MT group. The conflicting results between these two studies indicated further needs to explore the interaction effects of the timing of tDCS with MT to determine the optimal combination strategy.

The important factor to consider when examining the effects of combined tDCS with MT is the treatment outcomes, especially for outcomes that are related to daily activities. ADL such as the basic ADL and complex instrumental ADL (IADL) are essential for independent living and well-being of stroke patients. Therefore, restoring daily function should be one of the priority goals of stroke rehabilitation. However, the previous two studies only examined the effects of combined tDCS with MT on motor function [23, 24]. No studies to date have examined the timing-dependent effects of tDCS with MT on daily function in chronic stroke patients. Whether the timing of tDCS can affect restoration of daily function with MT remains uncertain.

In addition to daily function, investigating arm movement kinematics changes with respect to the timing of tDCS with MT is also critical for determining the optimal combination strategy. Movement kinematics of the arms can provide information of whether true behavioral changes or compensation strategies occur during training [25, 26]. However, the two previous studies included only clinical motor function measurements [23, 24]. While these clinical measurements can inform clinicians/researchers of motor function changes, they may not necessarily capture spatial and temporal characteristics of movement as well as motor control strategies changes after the combined interventions [26, 27]. Assessing movement kinematics changes with respect to the timing of tDCS with MT would help to unravel the benefits of combined approach on motor control of the paretic arm.

The purpose of this study was to examine the timing-dependent effects of tDCS with MT on daily function, upper extremity motor function and motor control in chronic stroke patients. The tDCS was applied sequentially prior to MT (i.e., sequentially combined tDCS with MT group, SEQ) or concurrently with MT (i.e., concurrently combined tDCS with MT, CON). The sham tDCS with MT was used as the control condition. In addition to motor function outcomes, we further included the ADL/IADL measurement and movement kinematics assessments. We hypothesized that the SEQ and COM groups would demonstrate differential improvements in daily function, motor function and motor control.

## Methods

### Participants

Individuals with stroke were recruited from medical centers in Taiwan. The inclusion criteria were (1) a first-ever unilateral stroke, (2) Age above 18 years old, (3) stroke onset more than 6 months, (4) Fugl-Meyer assessment (FMA) scores between 20 and 56, indicating moderate to mild impairments [28], (5) no severe muscle spasticity at the paretic arm at all joints (the Modified Ashworth Scale scores < 3), and (6) adequate cognitive function to follow instructions (the Mini Mental State Examination ≥24). The exclusion criteria were (1) participation in any drug or rehabilitation projects/experiments in the past 6 months, (2) had Botulinum toxin injections in the past 3 months, (3) severe vision or visual perception impairments (e.g., neglect and poor visual field) as assessed by the National Institutes of Health Stroke Subscale, (4) concomitant neurologic, neuromuscular or orthopedic conditions such as brain tumor and Parkinson’s disease, and (5) any contradictions to NIBS [29]. All participants gave their written informed consent before participating in this study. This study was approved by the Institutional Review Board of Chang Gung Memorial Hospital, Taoyuan, Taiwan. All study procedures were conducted in accordance with the Declaration of Helsinki.

### Design

**Fig. 1:**
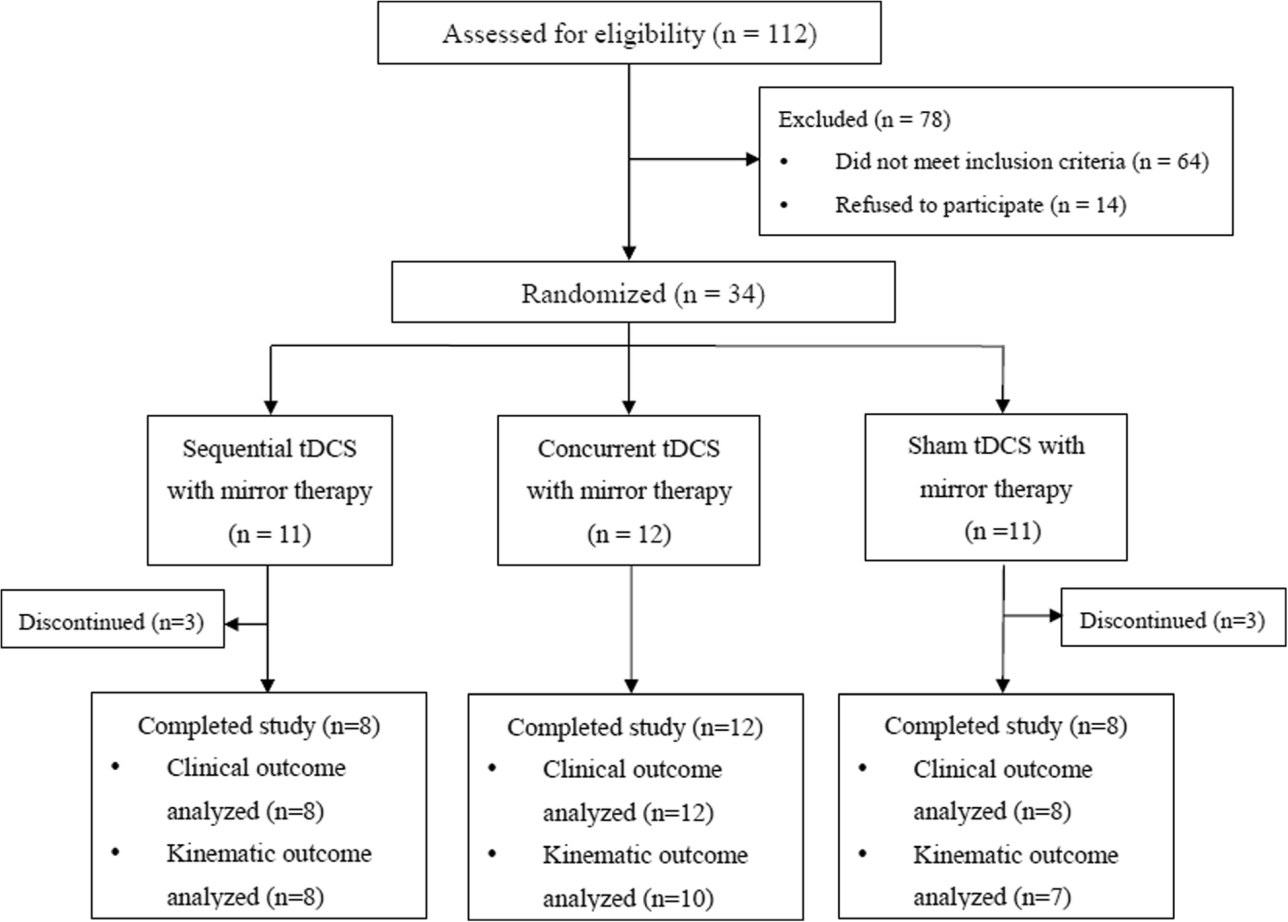
The CONSORT flow diagram

This study was a double-blinded, randomized controlled trial with pre-intervention and post-intervention assessments (Fig. 1). Participants were stratified based on their initial upper extremity impairment levels (FMA scores 20–35 vs. 36–56) [30] and randomly allocated to 3 groups: (1) sequential combination of tDCS with MT (SEQ), (2) concurrent combination of tDCS with MT (CON), and (3) sham tDCS with MT (SHAM). Randomization procedures were performed using a randomization table generated online (freely available at http://www.randomizer.org/).

Participants were assessed within 1 week before and after interventions by the same raters that were not involved in training and blinded to the purpose and group allocation of this study. These raters were trained by senior occupational therapists and the principal investigator of this study to ensure they performed the assessments in a uniformed and standardized way. Outcome measures included clinical and kinematic assessments. The clinical and kinematic assessments were performed on two separate days within 1 week before and after interventions to minimize feeling of fatigue during assessments.

### Intervention protocols

**Fig. 2:**
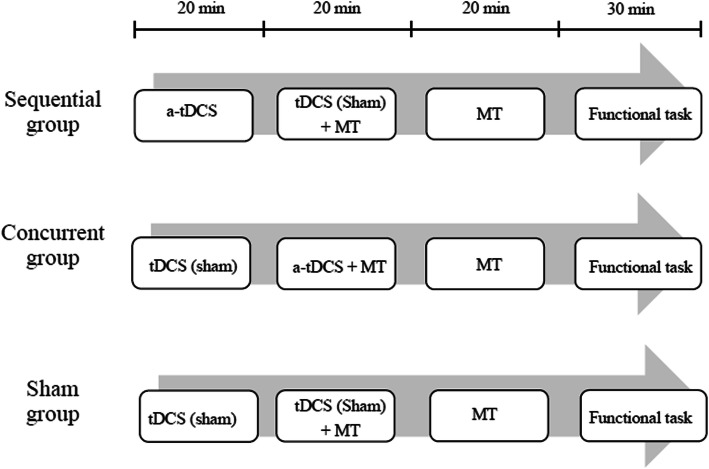
Experimental flow of the three intervention groups. Note: a-tDCS, anodal tDCS; MT, mirror therapy; tDCS, transcranial direct current stimulation

All participants received one of the three interventions for 90 min/day, 5 days/week, for 4 weeks. The intervention flow was illustrated in Fig. 2. The SEQ group received 20 min of anodal tDCS over the ipsilesional primary motor cortex (iM1) followed by 20 min of MT with sham tDCS and 20 min of MT alone. The CON group received sham tDCS during the first 20 min, followed by 20 min of MT concurrently with anodal tDCS on iM1 and 20 min of MT alone. The sham tDCS in the SEQ and CON groups was used to keep the tDCS setting consistent between SEQ and CON conditions to blind participants from group allocation and prevent them from noticing any differences in tDCS settings. For the SHAM group, the training/stimulation procedures were the same as those for the other two groups, except that there was only sham tDCS provided. There were 30 min of functional task practice performed after MT for all three groups.

#### tDCS protocol

A battery-driven direct current stimulator (neuroConn GmbH, Ilmenau, Germany) was used for delivering anodal tDCS. The anode electrode was placed over the iM1, which was the C3/C4 location of the international 10–20 electroencephalogram (EEG) electrode system; while the cathode electrode was placed on the contralesional supraorbital area. The duration of tDCS was 20 min. The size of the electrodes was 35 cm^2^, and the stimulation intensity was 2 mA, resulting in a current density of 0.057 mA/cm^2^ [31], which was well within the current safety limit [32]. For the sham tDCS, the stimulation intensity was first ramped up to 2 mA in 15 s and then ramped down to 0 within the next 30 s [33].

The tDCS protocols vary in the literature in terms of stimulation type, duration, intensity, and treatment sessions [7, 34]. The anodal tDCS on the iM1 and the cathodal tDCS on contralesional M1 have been applied in stroke patients with a range of 10 to 30 min of stimulation and a range of stimulation intensity between 1 to 2 mA [7, 34, 35]. The training sessions of combined tDCS with upper limb rehabilitation were between 14 to 30 sessions [7, 34, 35].

The tDCS protocol (2 mA of anodal tDCS for 20 min) used in this study was developed based on evidence in the literature [7, 34, 35]. We decided to use anodal tDCS because studies have shown that the anodal tDCS produced a more consistent modulation effects than the cathodal tDCS [36, 37]. The inter-individual variability was also lower using the anodal tDCS than the cathodal tDCS [36, 37]. Furthermore, anodal tDCS with an intensity of 2 mA and duration of 20 min was effective at increasing cortical excitability and producing long-lasting effects [37, 38]. Therefore, this tDCS protocol was implemented in the current study. Compared with the tDCS protocol of one previous study that examined the effects of combined tDCS with MT, the stimulation intensity and treatment sessions of our protocol were longer and only the lesioned cortex was stimulated in this study [23].

#### MT training and functional task practice

A mirror was placed in participants’ sagittal plane during MT. Participants were required to look at the reflection of non-paretic arm in the mirror, imagined it as the paretic arm and performed bilateral arm movements as simultaneously as possible. The MT training consisted of (1) intransitive movements, including distal and proximal arm/hand movements such as wrist extension-flexion, forearm pronation-supination and elbow flexion-extension, and (2) transitive movements, such as placing pegs in holes or flipping a card [13]. The functional task training focused on practice of daily activities that were meaningful and important for the participants. The contents of functional tasks were designed based on each participant’s main complaints about the tasks that were difficult to perform during daily activities. Examples of functional task practice included stabilizing a bowl with the paretic hand and scooping food with the non-paretic hand, grasping a cup with the paretic hand and bringing the cup toward the mouth, or wringing out water from a wet towel with both hands. Common daily objects, such as cups, bowls or towels were used for functional task practice based on the requirements of the tasks. Participants practiced three different kinds of functional tasks in 30 min [13].

### Outcome measures

#### Clinical outcome assessments

The Nottingham Extended Activities of Daily Living (NEADL) Scale was selected as the primary measure for evaluating the basic and instrumental ADL because it has been shown to have good psychometric properties (e.g. reliability and responsiveness) in stroke patients [39]. In addition, the NEADL does not have significant floor and celling effects in stroke patients and therefore it can be used in a wide range of stroke patients [40]. The NEADL consists of 22 items within four categories of daily activities, including mobility, kitchen, domestic, and leisure activities. The NEADL scale uses a 4-point rating scale. Higher scores indicate greater functional independence.

The Fugl-Meyer assessment scale of upper extremity (FMA) was used to assess sensorimotor function of upper limbs in individuals with stroke [41]. FMA consists of 33 movements with scores ranging from 0 to 66. Higher FMA scores indicate less impairment of the paretic arm. The validity and reliability of FMA are good to excellent in stroke patients [42].

#### Movement kinematic assessments

A unilateral reaching task was used to assess movement kinematics of the paretic arm and trunk. Participants were seated in front of a table with the seat height adjusted to 100% of the lower leg length. Participants placed their paretic hands at a marked starting point at the edge of table with their elbow flexed at 90 degrees. During the unilateral reaching task, participants were required to reach to press a doorbell that was placed along their midsagittal plane at a distance of 1.25 times the arm length (defined as the distance between the medial border of the axilla and the midpoint of the styloid processes of ulna and radius) as quickly as they could.

A seven-camera motion analysis system (VICON MX; Oxford Metrics Inc., Oxford, England) was used to capture motions of the paretic arm and the trunk. Reflective markers were attached to the 7th cervical vertebra (C7) and 4th thoracic vertebra (T4) spinal processes, mid sternum, bilateral clavicular head, acromion of the shoulder, anterior aspect of the upper arm midway between the acromion and lateral epicondyle, lateral epicondyle, ulnar and radial styloid processes, and the tip of thumb and index finger. Movements were recorded at 120 Hz and low-pass filtered at 5 Hz using a 2nd order Butterworth filter. A customized LabVIEW program (National Instruments Inc., Austin, TX) was used to process the kinematic data.

Kinematic outcome variables included the reaction time (RT), movement time (MT) and normalized total displacement (NTD). The hand marker (index finger) represented endpoint control and the sternal marker represented trunk control. Movement onset and offset were defined as the time point when the tangential velocity of the index finger/trunk rose above 5% and fell below 5% of the peak velocity of that trial [43]. RT was defined as the interval from the start signal to movement onset [44] and represented temporal efficiency of generation and planning of actions. Movement time was defined as the duration between movement onset and offset and represented movement efficiency. Hand NTD was defined as the path length of the hand, and normalized by the straight distance between the hand at the start position and the target for each participant. Trunk NTD was defined as the displacement of the sternal marker from the initial position, and normalized by the straight distance from the initial to end position for each participant. NTD represented movement straightness [45]. A smaller value of NTD indicated a more straight movement path.

### Statistical analysis

The chi-square test and the analysis of variance were used to examine baseline demographic and clinical characteristics of participants between groups. A paired t-test was used to compare differences from pre-intervention to post-intervention for each group. Analysis of covariance was used to evaluate differences of treatment effects between groups. The pre-intervention scores of each outcome were treated as the covariate to control for potential baseline differences. The effect size of partial eta squared (*η*^*2*^) was calculated for all outcome variables and represented the magnitude of changes between groups. *η*^*2*^ greater than 0.138 represented a large effect. *η*^*2*^ greater than 0.059 represented a moderate effect, and *η*^*2*^ greater than 0.01 represented a small effect [46]. The effect size *d* was also calculated for all outcomes and represented the magnitude of changes from pre- to post-intervention in each group [46]. Data were analyzed using SPSS 19.0 (IBM Corp., Armonk, NY). The alpha level was set at 0.05.

## Results

Thirty-four participants were enrolled in the study. Three participants in the SEQ group and three participants in the SHAM group did not complete interventions due to unexpected family events, lack of transportation, work responsibilities, childcare commitments and no-show. Therefore, twenty-eight participants completed all training sessions. There were eight participants in the SEQ group, twelve participants in the CON group and eight participants in the SHAM group (Fig. 1).

Participants’ baseline demographics and clinical characteristics did not significantly differ between groups (Table 1). All twenty-eight participants completed clinical assessments (i.e., FMA and NEADL) at pre-intervention and post-intervention. Among them, three participants (two participants in the CON group and one participant in the SHAM group) could not perform the unilateral reaching task correctly for kinematic assessments. To minimize potential measurement errors, these three participants did not undergo kinematic assessments. The kinematic data included 25 participants (Fig. 1).

**Table 1:** Demographic and clinical characteristics of the study participants

Variables	SEQ (*N* = 8)	CON (*N* = 12)	SHAM (*N* = 8)	*Statistic*^a^	*P*
Age (year)	60.18 ± 4.84	52.04 ± 8.68	56.45 ± 9.88	2.43	0.11
Gender (Male/Female)	5/3	8/4	8/0	3.78	0.15
Side of lesion (Right/Left)	2/6 [45]	5/7	0/8	4.44	0.11
Onset time (months)	19.63 ± 12.28	21.92 ± 11.83	38.13 ± 36.98	1.75	0.2
Hemorrhagic/Ischemic Stroke	2/6	5/7	1/7	2.07	0.36
FMA	37.50 ± 14.95	36.33 ± 7.91	30.75 ± 9.16	0.95	0.4
MMSE	28.25 ± 1.67	28.83 ± 2.12	27.38 ± 2.56	1.11	0.35
MAS	0.39 ± 0.17	0.50 ± 0.28	0.61 ± 0.24	1.64	0.22

^a^Statistic associated with the chi-square test for categorical variables and with the analysis of variance for continuous variables. Note: *SEQ* Sequential group, *CON* Concurrent group, *SHAM* Sham group, *FMA* Fugl-Meyer assessment scale of upper extremity, *MMSE* Mini-Mental State Examination, *MAS* Modified Ashworth Scale. Value is presented as mean ± standard deviation (SD)

Table 2 summarizes the descriptive and inferential statistics for within-group and between-group comparison of clinical and kinematic outcomes. For within group comparison, significant improvements were found in the NEADL scores from pre-intervention to post-intervention in the SEQ (*t* = 3.18, *P* = 0.02, *d* = 0.84) and CON (*t* = 3.6, *P* = 0.004, *d* = 0.22) groups. However, no changes were found in the NEADL scores from pre-intervention to post-intervention in the SHAM group (*t* = 0.47, *P* = 0.65*, d* = 0.05). All three groups had significant increase in the FMA scores from pre-intervention to post-intervention (SEQ, *t* = 4.36, *P* = 0.003, *d* = 0.39; CON, *t* = 3.15, *P* = 0.01, *d* = 0.54; SHAM, *t* = 3.15, *P* = 0.02, *d* = 0.38). For the kinematic outcomes, significant improvement was demonstrated in the index movement time of the SEQ group from pre-intervention to post-intervention (*t* = − 2.38, *P* = 0.04, *d* = 0.3). No differences were found in other kinematics variables from pre-intervention to post-interventions in the three groups (*t* = − 2.18 to 1.63, *P* = 0.07 to 0.98, *d* < 0.001 to *d* = 1.01).

**Table 2:** Descriptive and inferential statistics of clinical and kinematic outcomes

	Pretreatment			Posttreatment			Between-group			Within-group								
										SEQ			CON			SHAM		
**Clinical Variables**	SEQ	CON	SHAM	SEQ	CON	SHAM	*F*	*P*	*η*^2^	*t*	*P*	*d*	*t*	*P*	*d*	*t*	*P*	*d*
	Mean ± SD	Mean ± SD	Mean ± SD	Mean ± SD	Mean ± SD	Mean ± SD												
NEADL	28.00 ± 7.82	33.42 ± 15.63	23.88 ± 8.82	35.75 ± 10.48	37.00 ± 16.46	24.38 ± 9.98	4.69	0.02*	0.28	3.18	0.02*	0.84	3.60	0.004*	0.22	0.47	0.65	0.05
FMA	37.50 ± 14.95	36.33 ± 7.91	30.75 ± 9.16	43.25 ± 14.34	41.67 ± 11.41	34.50 ± 10.46	0.27	0.77	0.02	4.36	0.003*	0.39	3.15	0.01*	0.54	3.15	0.02*	0.38
**Kinematic Variables**																		
Trunk RT (sec)	0.29 ± 0.12	0.36 ± 0.14	0.29 ± 0.10	0.31 ± 0.78	0.27 ± 0.59	0.29 ± 0.07	0.53	0.6	0.05	0.56	0.59	0.04	−1.58	0.15	0.21	0.09	0.93	< 0.001
Index RT (sec)	0.47 ± 0.17	0.53 ± 0.12	0.52 ± 0.20	0.47 ± 0.16	0.44 ± 0.18	0.40 ± 0.09	0.30	0.74	0.03	−0.03	0.98	< 0.001	−1.25	0.24	0.59	− 1.28	0.25	0.77
Trunk MT (sec)	2.40 ± 0.83	2.17 ± 0.69	2.27 ± 1.24	1.63 ± 0.36	2.09 ± 1.14	1.72 ± 0.58	0.74	0.49	0.07	−2.18	0.07	1.2	−0.23	0.83	0.08	−1.01	0.35	0.57
Index MT (sec)	2.30 ± 0.80	1.92 ± 0.92	1.88 ± 1.14	1.50 ± 0.37	2.00 ± 1.19	1.66 ± 0.50	0.9	0.42	0.08	−2.38	0.04*	0.3	0.21	0.84	0.08	−0.42	0.69	0.25
Trunk NTD	1.25 ± 0.35	2.35 ± 3.33	1.09 ± 0.04	1.08 ± 0.48	1.12 ± 0.89	1.16 ± 0.19	0.66	0.53	0.06	−1.29	0.24	0.4	−1.16	0.28	0.5	0.82	0.45	0.51
Index NTD	1.99 ± 0.55	1.76 ± 0.28	1.46 ± 0.19	1.55 ± 0.28	1.68 ± 0.26	1.84 ± 0.51	0.15	0.87	0.01	−1.63	0.15	1.01	−0.59	0.57	0.3	1.63	0.16	0.98

*SEQ* Sequential group, *CON* Concurrent group, *SHAM* Sham group, *FMA* Fugl-Meyer Assessment Scale of Upper extremity, *NEADL* Nottingham Extended Activities of Daily Living Scale, *RT* reaction time, *MT* movement time, *NTD* normalized total displacement. *P≦0.05

**Fig. 3:**
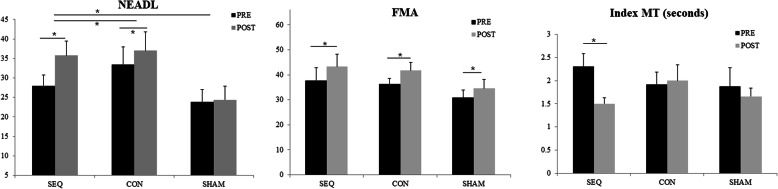
Changes of NEADL, FMA and index MT in the three intervention groups. Note: NEADL, Nottingham Extended Activities of Daily Living scale; FMA, Fugl-Meyer Assessment scale of Upper Extremity; MT, movement time (seconds). Data is presented as mean ± standard error. SEQ, Sequential group; CON, Concurrent group; SHAM, Sham group

For between group comparison, significant differences were found in the NEADL scores (*F*_*(2,25)*_ = 4.69, *P* = 0.02, *η*^*2*^ = 0.28). Post hoc analyses showed that the SEQ group had significantly greater increase in the NEADL scores than the CON (*P* = 0.05) and the SHAM (*P* = 0.006) groups. However, no differences were found in the NEADL scores between the CON and SHAM groups (*P* = 0.24). There were no differences in the FMA scores (*F*_*(2,25)*_ = 0. 27, *P* = 0.77, *η*^*2*^ = 0.02) and kinematic variables (*F*_(2,22)_ = 0.15 to 0.9, *P* = 0.42 to 0.9, *η*^*2*^ = 0.01 to 0.08) between groups. Fig. 3 illustrates the significant changes in the clinical (i.e., NEADL and FMA) and kinematic (i.e., index finger movement time) outcomes.

## Discussion

To our knowledge, this study was the first to examine timing-dependent effects of tDCS with MT on daily function and movement control in participants with chronic stroke. We found greater improvements in overall ADL/IADL function in the SEQ group compared to the CON and SHAM groups, indicating that the timing of tDCS may affect restoration of basic and complex ADL abilities for independent living in the community. Furthermore, kinematic analyses revealed that the index movement time significantly reduced only in the SEQ group, but not the CON and SHAM groups. This result indicated that the SEQ group could move the paretic hand more efficiently during unilateral reaching tasks after interventions. By contrast, the CON and SHAM group did not show such improvements in motor control of paretic hands after interventions. All three groups had significant improvements in motor function.

Consistent with our hypothesis, we found timing-dependent effects of tDCS with MT. In particular, this timing-dependent effect was exhibited in ADL/IADL function, but not clinical motor function. Applying tDCS prior to MT led to greater benefits on daily function than applying tDCS concurrently with MT. Compared with motor tasks, the ADL/IADL tasks often involve higher levels of motor-cognitive and perceptual processes and require the ability to transfer the learned skills to daily tasks [47–49]. In this study, during the SEQ condition, the anodal tDCS may serve as a priming stimulus to enhance activity of the lesioned cortex. This priming stimulus would create a favorable/excitable environment of the brain which is beneficial for activating higher-order motor-cognitive processes during the consecutive MT [50–52]. This activated brain state accompanied by repetitive practice of MT might facilitate the cognitive-motor relearning processes and enhance generalization of learned motor skills [7, 53]. By contrast, applying tDCS concurrently with MT might generate motor/cognitive interference during the MT practice, and consequently affect the cognitive-motor relearning processes and generalization of learned skills to daily activities [53–55]. Studies have also reported that delivering tDCS concurrently with motor/cognitive trainings interfered or abolished the modulatory effects of tDCS [55–58]. Our results were in line with these previous findings suggesting that the effects of tDCS with MT may not be simply additive, and that applying tDCS prior to MT might be a potentially beneficial approach for improving ADL/IADL function more than concurrent tDCS with MT in chronic stroke patients.

Contrary to our hypothesis, motor function improved to a similar extent between groups. These results indicated that there might be potentially no timing-dependent effects of tDCS with MT, and combination of tDCS with MT either sequentially or concurrently did not yield to additional benefits on motor function than MT alone. Though surprising, these results were similar to the findings of no add-on effects of tDCS with motor-imagery based modality (e.g., brain-computer interface, BCI) or motor training on clinical motor function in several previous studies [59–61]. A recent systematic review of randomized controlled trials of tDCS also did not find additional benefits of tDCS on improving motor function (i.e., FMA scores), but rather benefits of tDCS on enhancing ADL capacity [62].

However, our finding of similar levels of motor function improvements between the SEQ and CON groups was contrary to the results of one previous study that examined the effects of combined tDCS with MT [23]. In that study, the concurrent-tDCS group showed greater improvements in motor function than that of prior-tDCS and sham groups. Nevertheless, our study protocol was different from those of the previous study in several ways. First, the stimulation paradigm was different. We stimulated the ipsilesional primary motor cortex while the previous study modulated both ipsilesional and contralesional primary motor cortex. Second, the stimulation intensity was higher (i.e., 2 mA) in our study than the previous study (i.e., 1 mA). Third, our participants received longer duration of interventions (i.e., 20 sessions) than that of previous study (i.e., 10 sessions). It is possible that these differences in tDCS and intervention protocols resulting in the differential findings on motor function between our study and the previous study [54]. This result also indicated that tDCS parameters such as intensity and duration as well as modules of stimulation might affect the interaction effects of tDCS with MT on motor function, and these factors need to be carefully considered during clinical application. Future studies could examine the timing-dependent effects of tDCS with MT using different tDCS stimulation parameters and intervention protocols to optimize treatment benefits on motor function.

Another important question of this study was whether there would be timing-dependent effects of tDCS with MT on movement control of paretic upper extremity. In addition to clinical outcomes, the kinematic assessments were also employed to objectively track changes of spatial and temporal control of upper extremity. We found that movement efficiency of the paretic hand significantly improved in the SEQ group after interventions. This finding was consistent with the evidence reported by Giacobee et al. (2013) [63]. In their study, wrist movement kinematics improved only when tDCS was applied sequentially prior to robotic training, but not concurrently with training. As a result, it is possible that the order of tDCS with neurorehabilitation may influence restoration of distal arm movement control such as the wrist and hand. In particular, sequentially combined tDCS with MT may have potentials to improve distal hand control. Our study also indicated that kinematic assessments were sensitive to the timing-dependent effects of tDCS with MT on motor control of the paretic arm because arm performance changes were revealed by kinematic analyses, but not clinical measures. Hence, we recommended future studies to include kinematic assessments to unravel the true add-on effects and timing-dependent effects of tDCS with neurorehabilitation interventions regarding paretic arm performance.

Five potential limitations should be considered. First, in view of the clinical characteristics of the enrolled participants, our findings may be applicable only to individuals with mild-to-moderate impairment at the chronic stage. Second, although our study demonstrated timing-dependent effects of tDCS with MT on daily function and arm kinematics, it may also be beneficial for future studies to include other outcome measures such as neurophysiological outcomes (e.g., Motor evoked potentials) and neuroimaging outcomes (i.e., brain imaging data). This will help to determine whether the timing of tDCS with MT would affect recovery of neural mechanisms. Third, there were no follow-up assessments. Future studies could evaluate whether the timing-dependent effects of tDCS with MT on daily function and hand movement control would be maintained during the follow-up period. Fourth, the functional task training performed in this study was individualized to each participant although the training duration, frequency and categories were standardized for the three groups. Future studies could examine whether the contents of functional task practice would affect treatment effects. Fifth, differential improvements were found in some kinematic outcomes between the three groups although these between-group differences did not reach statistically significant. For example, the normalized total displacement of the index finger and the trunk movement time reduced to the greater extent in the SEQ than the CON and SHAM groups, suggesting potential improvements in the spatial and temporal control of the hand and the trunk primarily in the SEQ group. However, these differences did not reach statistically different between groups, possibly due to the small sample size of this pilot study. Future studies could recruit more participants to determine whether the spatial and temporal kinematic outcomes (i.e., trunk movement time and index movement displacement) would be affected by the timing of tDCS with MT in chronic stroke patients.

## Conclusion

Our study demonstrated potential timing-dependent effects of tDCS with MT on daily function and motor control of paretic hand in chronic stroke patients. Sequentially applying tDCS prior to MT could enhance ADL/IADL function more than applying tDCS concurrently with MT or sham stimulation. Hand movement efficiency also improved in the sequentially combined group. These findings indicated that sequentially combining tDCS prior to MT might be a potentially useful strategy especially for restoration of daily function and paretic hand control for chronic stroke patients, thus may be considered in future clinical application. Further studies with a larger sample size are warranted.

## Data Availability

The datasets used and/or analyzed during the current study are available from the corresponding author on reasonable request.

## Abbreviations

NIBS: Non-invasive brain stimulation
tDCS: Transcranial direct current stimulation
MT: Mirror Therapy
ADL: Activities of daily living
IADL: Instrumental activities of daily living
SEQ: Sequentially combined tDCS with MT
CON: Concurrently combined tDCS with MT
SHAM: Sham tDCS with MT
iM1: Ipsilesional primary motor cortex
EEG: Electroencephalogram
NEADL: Nottingham Extended Activities of Daily Living
FMA: Fugl-Meyer assessment scale of upper extremity
C7: The 7th cervical vertebra
T4: The 4th thoracic vertebra
RT: Reaction time
MT: Movement time
NTD: Normalized total displacement
